# Editorial: The Neural Signatures of Plasticity in Developmental and Early Acquired Speech, Language and Reading Disorders

**DOI:** 10.3389/fnhum.2021.771567

**Published:** 2021-10-22

**Authors:** Guadalupe Dávila, Heidi M. Feldman, Diana López-Barroso

**Affiliations:** ^1^Cognitive Neurology and Aphasia Unit, Centro de Investigaciones Médico-Sanitarias, University of Malaga, Malaga, Spain; ^2^Instituto de Investigación Biomédica de Málaga—IBIMA, Malaga, Spain; ^3^Department of Psychobiology and Methodology of Behavioural Sciences, Faculty of Psychology and Speech Therapy, University of Malaga, Malaga, Spain; ^4^Language Neuroscience Research Laboratory, Faculty of Psychology and Speech Therapy, University of Malaga, Malaga, Spain; ^5^The Developmental-Behavioral Pediatrics Research Group, Division of Developmental-Behavioral Pediatrics, Department of Pediatrics, School of Medicine, Stanford University, Stanford, CA, United States

**Keywords:** plasticity, childhood, neurodevelopment, brain damage, language disorders, reading disorders

The brain has an astonishing capacity to change, allowing both learning throughout life and recovery from brain damage. However, different brain injuries during the development and maturation of speech, language, and reading functions may impact the neurodevelopmental outcomes by interfering with adaptive neural plasticity. Therefore, studying children and adolescents born preterm, suffering from congenital deafness, developmental dyslexia (DD), brain malformations or acquired damage is paramount to gain further insights into the neuroplastic mechanisms operating in the developing brain. In this Research Topic, we assemble a collection of nine studies that apply the most advanced neuroimaging technology in different populations to bring new vitality and insight to this complex topic.

The article by Demir-Lira et al. shows that early language experience may play a relevant role in developing brain structure. These researchers describe a longitudinal study spanning 15 years and reveal, for the first time, that features of parental language input before school predict the level of change in cortical thickness between 5 and 12 years later. An essential role of deprivation on white matter tract maturation is shown by Cheng et al.'s study, which evaluated children with different early language experiences, including deaf native signers of American Sign Language (ASL), hearing L2 signers of ASL, and children with limited early language experience. This research provides evidence that the sensory modality of early language experience does not affect the structure of the dorsal and ventral white matter language pathways as revealed using DTI-Tractography. Conversely, early deprivation of linguistic experience of any kind does affect the structure of the left arcuate fasciculus.

Neuroplasticity is also the prevailing model for explaining the neuroimaging correlates of recovery in children with prenatal or perinatal brain injuries that show normal development after an initial delay. In one study, Bruckert et al. demonstrate that at the age of 8 years, there is no difference in reading performance between children born full-term and pre-term, nor there are differences in the microstructural properties of dorsal and ventral white matter pathways related to reading between the two groups. However, the microstructure of these pathways forecasts later reading outcomes only in children born full-term. By contrast, reading in children born pre-term may rely on alternative brain routes or on a broader set of cognitive skills related to more extensive brain networks (e.g., executive functions) to achieve proficiency in reading performance. In another study, Northam et al. evaluate oromotor and speech production abilities while structural and functional magnetic resonance imaging (MRI) was acquired in adolescents born prematurely. They detected persistent subclinical oromotor control difficulties in 31% of the sample, yet no speech disorders were found in any participants. However, the authors found altered microstructure in the impaired group within the part of the corticospinal tract associated with the control of the lips, tongue and larynx, as well as greater recruitment of the perisylvian areas in the right hemisphere, suggesting that the right hemisphere may compensate for early damage in left-hemispheric areas that may be important for normal speech production.

Neuroplasticity may be inefficient in cases of language deficits associated with bilateral developmental brain malformations. The multimodal study of Berthier et al. describes, for the first time, a case of developmental dynamic dysphasia in an adolescent showing bilateral perisylvian cortical anomalies (left greater than right) together with atypical configuration of white matter tracts, including the corpus callosum. Dynamic dysphasia, characterized by deficits in verbal fluency and sentence generation, probably arose from a limited capacity of the malformed right hemisphere to take over left hemisphere language deficits and from altered interhemispheric interaction. A second case of developmental dynamic dysphasia has recently been described by Barker et al. (2021) in an adult patient related to a congenital malformation of the rhombencephalon and corpus callosum.

While language deficits associated with developmental brain malformations persist throughout life, focal brain lesions acquired during childhood can improve when treatments based on neuroscience principles are used. Indeed, language recovery can even be boosted by using combined interventions. Dávila et al. report the case of a 9-year-old girl with chronic anomic aphasia after traumatic brain injury in the left temporoparietal cortex. The patient received a novel combined therapy to promote brain plasticity, involving a cholinergic agent (donepezil) alone and in combination with intensive anomia therapy. Treatment with donepezil alone was enough to improve executive and language deficits, while adding the language therapy boosted the gains that remained after the washout period. For the first time, this study demonstrated that donepezil, a drug effective for treating adult aphasia (Berthier et al., 2006) administered alone and in combination with intensive naming therapy is well-tolerated, safe and effective in treating childhood aphasia.

DD, one of the most prevalent developmental disorders, is a selective deficit of reading abilities not explained by a deficit of general intelligence or educational opportunities (Peterson and Pennington, 2012). Causative factors, both biological or cognitive, have been proposed, but the precise characterization of the etiology and different profiles of dyslexia must be conclusively demonstrated. On studying DD, Horowitz-Kraus et al. highlight the role of executive functions associated with the activity of medial prefrontal brain areas such as the anterior cingulate cortex (ACC) using proton magnetic resonance spectroscopy. Specifically, they show that processing speed exerts a protective action against dyslexia. Furthermore, this cognitive function in female dyslexics correlates with low levels of metabolites associated with myelination and connectivity in the ACC. Cao et al. examine brain mechanisms underlying visuo-orthographic processing through lexical and perceptual tasks in children with DD. Brain activation analysis revealed decreased activation in the left precuneus associated to both visual and orthographic deficits, likely reflecting a deficit in visual attention; while Psycho-Physiological Interaction (PPI) analysis revealed task-specific alterations in functional connectivity. An alternative view is provided by Zakopoulou et al., who address DD from a multifactorial causal model to explore the link between brain asymmetries, personality traits, cognitive skills, specific gene expression profiles, neuroplasticity, and stress in an adult diagnosed with DD. This single case study suggests a stress-related dyslexia endophenotype characterized by asymmetries in the amygdala and cerebellum, altered stress response and personality traits such as difficulties coping with intense emotional situations.

In conclusion, these papers demonstrate that early brain injuries contribute to but are not the sole predictors of language outcomes. Taken together, we see the importance of brain structure to language function, not only classic brain areas but also other areas. We learned that children born preterm learn to speak despite subclinical oromotor abnormalities and read despite different patterns of white matter association. We documented the importance of experience for brain development, including the positive impact of parental input and the negative effects of deprivation. We documented that a medication affecting neural transmitters can boost recovery after traumatic injury. Thus, we hope that this Research Topic will help resolve long-standing questions on plasticity during the early stages of development and propel new ideas for future studies.

## Author Contributions

GD and DL-B contributed to the design of the work and drafted the Editorial. HF revised the draft for important intellectual content and contributed with the interpretation of the work. GD, DL-B, and HF approved the final version to be published. All authors contributed to the article and approved the submitted version.

## Funding

GD and DL-B have been supported by a grant from the Spanish Ministry of Science and Innovation, Health Institute Carlos III (PI16/01514.). DL-B has been funded by an I+D+i Project, Andalusia and European Union Funds (FEDER) (UMA18-FEDERJA-221).

## Conflict of Interest

The authors declare that the research was conducted in the absence of any commercial or financial relationships that could be construed as a potential conflict of interest.

## Publisher's Note

All claims expressed in this article are solely those of the authors and do not necessarily represent those of their affiliated organizations, or those of the publisher, the editors and the reviewers. Any product that may be evaluated in this article, or claim that may be made by its manufacturer, is not guaranteed or endorsed by the publisher.
